# Extraction of Pathogenesis-Related Proteins and Phenolics in Sauvignon Blanc as Affected by Grape Harvesting and Processing Conditions

**DOI:** 10.3390/molecules22071164

**Published:** 2017-07-12

**Authors:** Bin Tian, Roland Harrison, James Morton, Marlene Jaspers, Simon Hodge, Claire Grose, Mike Trought

**Affiliations:** 1 Faculty of Agriculture and Life Sciences, Lincoln University, Lincoln 7647, New Zealand; roland.harrison@lincoln.ac.nz (R.H.); james.morton@lincoln.ac.nz (J.M.); marlene.jaspers@lincoln.ac.nz (M.J.); simon.hodge@lincoln.ac.nz (S.H.); 2 Marlborough Research Centre, New Zealand Institute for Plant and Food Research Ltd., Blenheim 7240, New Zealand; claire.grose@plantandfood.co.nz (C.G.); mike.trought@plantandfood.co.nz (M.T.)

**Keywords:** bentonite, harvesting, pathogenesis-related (PR) proteins, phenolics, processing

## Abstract

Thaumatin-like proteins (TLPs) and chitinases are the two main groups of pathogenesis-related (PR) proteins found in wine that cause protein haze formation. Previous studies have found that phenolics are also involved in protein haze formation. In this study, Sauvignon Blanc grapes were harvested and processed in two vintages (2011 and 2012) by three different treatments: (1) hand harvesting with whole bunch press (H-WB); (2) hand harvesting with destem/crush and 3 h skin contact (H-DC-3); and (3) machine harvesting with destem/crush and 3 h skin contact (M-DC-3). The juices were collected at three pressure levels (0.4 MPa, 0.8 MPa and 1.6 MPa), some juices were fermented in 750 mL of wine bottles to determine the bentonite requirement for the resulting wines. Results showed juices of M-DC-3 had significantly lower concentration of proteins, including PR proteins, compared to those of H-DC-3, likely due to the greater juice yield of M-DC-3 and interactions between proteins and phenolics. Juices from the 0.8–1.6 MPa pressure and resultant wines had the highest concentration of phenolics but the lowest concentration of TLPs. This supported the view that TLPs are released at low pressure as they are mainly present in grape pulp but additional extraction of phenolics largely present in skin occurs at higher pressing pressure. Wine protein stability tests showed a positive linear correlation between bentonite requirement and the concentration of chitinases, indicating the possibility of predicting bentonite requirement by quantification of chitinases. This study contributes to an improved understanding of extraction of haze-forming PR proteins and phenolics that can influence bentonite requirement for protein stabilization.

## 1. Introduction

Proteins remaining in white wine after fermentation may result in unattractive haze formation. It is now well-established that the principle soluble haze-forming proteins in white wine are pathogenesis-related (PR) proteins, which are derived from grape berries [1,2] and their concentration in grapes can be affected by fungal infection [3,4] and ultraviolet radiation [5]. In general, fungal disease such as downy mildew and powdery mildew would lead to increased level of PR proteins in grape berries, but the decrease of PR proteins in grapes infected by *Botrytis cinerea* and resultant juice had been observed, which is possibly due to the secretion of proteases by *B. cinerea* [4,6,7]. The two major soluble PR proteins found in wine are thaumatin-like proteins (PR-5 family) and chitinases (PR-3 family) with molecular masses of 24 kDa and 32 kDa, respectively [8]. Both thaumatin-like proteins (TLPs) and chitinases have been found in Sauvignon Blanc grape skin and pulp [9]. Other PR proteins found in grapevines include osmotins (PR-5 family), β-1, 3-glucanases (PR-2 family) and the PR-10 proteins [10,11,12]. These PR proteins are involved in defense mechanisms against pathogen attack as well as resulting from wounding and certain abiotic stresses [12,13,14]. They are highly resistant to low pH and proteolytic degradation [15], and thus survive fermentation and potentially form undesirable hazes in wines [16].

The reduction in protein haze as a result of polyvinyl polypyrrolidone (PVPP) fining of commercial wines suggested that phenolics may play a modulating role in haze formation [17]. The observation of several phenolic compounds in natural wine protein haze by Esteruelas et al. [18] also indicated the involvement of phenolics in wine protein haze formation. The polyphenols could participate in the aggregation and precipitation of wine proteins through the formation of hydrogen bonds or the hydrophobic interactions with proteins [1,19,20,21,22]. In a recent study, Gazzola et al. [23] used reconstitution experiments to demonstrate the roles of phenolics and polysaccharides as well as of four TLPs isoforms and chitinases in white wine haze formation. The results showed that: (1) chitinases were the proteins most prone to aggregate and form the largest particles even in the absence of phenolics and polysaccharides; and (2) TLP isoforms varied in susceptibility to haze formation and in their interactions with polysaccharides and phenolics. The authors also suggested that polysaccharides are able to reduce the formation of protein-polyphenol aggregates by interacting with phenolics, but this inhibiting effect of polysaccharides is likely governed by the structure and the chemicophysical characteristics of individual wine proteins. The results of TLP isoforms displaying different hazing potentials were also supported by a recent structural study of TLPs from white wine [24]. Therefore, it is necessary to take into account the phenolics co-extracted with proteins from grapes to evaluate eventual wine protein stability.

The concentration of PR proteins and phenolics remaining in wine is largely determined by their concentrations in pre-fermentation juice which in turn is determined by the extraction of these compounds from grape berries. Our previous study [5] showed the concentration of PR proteins in juice is predominantly determined by their concentration in grape pulp, but factors (e.g., ultraviolet radiation) that influence the concentration of phenolic substances/tannins could modulate the final concentration of PR proteins in juice due to the interactions between proteins and phenolics during the juicing process. Thus, harvesting and grape processing practices are important factors. The phenolic content of wine produced from grapes harvested by machine is greater than that from grapes harvested by hand [25], possibly because machine harvesting facilitates greater extraction of phenolics from grapes. Skin contact has also been found to increase the phenolic concentration of Sauvignon Blanc juice [26] and in analysis of white wines [27]. In addition, Darias-Martín et al. [28] compared two pressing processes in Spanish white wine production and found that maceration resulted in higher levels of phenolics in the must than direct pressing.

Despite their importance, studies on the influence of grape harvesting and processing techniques on extraction of haze-forming PR proteins are very limited. An early study by Pocock et al. [29] suggested that machine harvesting coupled with long distance transport (about 20 h) could be expected to increase juice protein concentration (including PR proteins) because of greater extraction of proteins from the skin of the broken berries during transport. Oxidation during transport resulted in reduced phenolic concentration but had little influence on protein concentration [30]. In other winemaking regions, transport times from vineyard to winery are much shorter and this may influence extraction during transport.

The aim of this study is to determine the influence of various combinations of commercial-scale grape harvesting and processing conditions, including skin contact, on the extraction of haze-forming PR proteins and phenolics, and the consequent impact on wine heat stability. In addition, the effects of factors known to influence phenolic extraction were of particular interest.

## 2. Results

### 2.1. Analysis of Total Soluble Solids and Acidity in Grapes Harvested in Three Blocks

The three different grape processing treatments in each block are described in Table 1. Grapes from Dillons Point vineyard in 2011 (2011DP) and in 2012 (2012DP) were harvested at approximately 20 °Brix, and grapes from Ben Morven vineyard in 2012 (2012BM) were harvested at approximately 21 °Brix. The corresponding juices from the different processing treatments across these blocks showed the °Brix values ranging from 19.2 to 21.7, the pH values ranging from 2.94 to 3.08, and titratable acidity (TA) values ranging from 11.1 g/L to 14.6 g/L, respectively (Table 2). The pH values of grape juices were similar within treatments across blocks, but the titratable acidity of juices from 2012DP was higher than those from 2011DP. This was likely due to variation in acid composition due to difference in environmental factors over two vintages.

### 2.2. Analysis of Protein and Phenolic Content in Juices from Three Blocks

Different harvesting methods, grape processing conditions and pressing pressure resulted in considerable differences in juice yield, ranging from 13.5% to 73% (Table S1). The corresponding statistical analysis is shown in Table 3. Overall, the highest juice yield was produced by M-DC-3 with 64.5%, and the lowest by H-WB with 39.3%. As expected, the highest juice yield was produced using the highest extraction pressure of 0–1.6 MPa (66.3%), and the lowest juice yield at the lowest extraction pressure of 0–0.4 MPa (39.4%). Thus, the highest juice yield achieved across the three blocks was obtained using a combination of M-DC-3 and 0–1.6 MPa at 71.9%. The lowest juice yield obtained was 15.3% with a combination of H-WB and 0–0.4 MPa.

The concentration of proteins, phenolics, TLPs and chitinases in juices across the three blocks ranged from 51.5 to 224.4 mg/L, from 161.6 to 275.8 mg/L, from 55.3 to 152.0 mg/L, and from 28.7 to 110.0 mg/L, respectively (Table S1). Significant differences in concentration of proteins (*p* < 0.01), phenolics (*p* < 0.01), TLPs (*p* < 0.01), and chitinases (*p* < 0.05) were observed among the different processing treatments (Table 3). The concentration of proteins in juices from M-DC-3 was significantly lower than that from H-DC-3 but similar to that from H-WB. The lowest concentration of phenolics and TLPs was observed in juices from H-WB and M-DC-3, respectively. The concentration of chitinases in juices from H-WB was significantly lower than that from H-DC-3 but similar to that from M-DC-3. There were no significant differences observed in the concentrations of proteins, phenolics and chitinases of juices obtained by applying different pressing pressures. The concentration of TLPs in juices was influenced by pressing pressure (*p* < 0.05) but not in a straightforward manner, being lowest in the high pressure extraction (101.0 mg/L) and highest in the medium pressure extraction (107.6 mg/L), with the low pressure extraction producing an intermediate value (104.3 mg/L). However, the range in concentrations between these treatments was small.

The total extraction of proteins, phenolics, TLPs and chitinases in juices has been calculated by multiplying the corresponding concentration and juice yield, and expressed as mg/kg of grapes (Table S1). Both processing treatment and pressing pressure resulted in significant differences in the extraction of proteins, phenolics, TLPs, and chitinases in corresponding juices (Table 3). Juices from the H-WB treatment had the lowest extraction of proteins (*p* < 0.001), phenolics (*p* < 0.001), TLPs (*p* < 0.001) and chitinases *(p* < 0.001). Juices obtained at the lowest pressure of 0–0.4 MPa also showed the lowest extraction of proteins (*p* < 0.001), phenolics (*p* < 0.001), TLPs (*p* < 0.001) and chitinases (*p* < 0.001). Thus, a combination of H-WB and 0–0.4 MPa (*p* < 0.05) consequently resulted in the lowest amount of extracted proteins (18.5 mg/kg of grapes), phenolics (32.9 mg/kg of grapes), TLPs (15.4 mg/kg of grapes) and chitinases (10.3 mg/kg of grapes).

### 2.3. Analysis of Juices and Wines from the Separate Pressing Fractions in 2012DP

Results of analysis of proteins and phenolics of juices and wines from three separate pressing fractions (0–0.4 MPa, 0.4–0.8 MPa and 0.8–1.6 MPa) of 2012DP are presented in Table S1. The concentration of proteins, phenolics, TLPs and chitinases in juices ranged from 32.8 to 205.5 mg/L, from 224.6 to 300.2 mg/L, from 108.2 to 160.2 mg/L, and from 94.1 to 116.2 mg/L, respectively. The concentration of proteins, phenolics, TLPs and chitinases in wines ranged from 60.2 to 120.2 mg/L, from 202.7 to 269.4 mg/L, from 53.0 to 104.8 mg/L, and from 24.1 to 62.7 mg/L, respectively. Among the different processing treatments, significant differences were only observed for the concentration of phenolics in juices (*p* < 0.001), with the H-WB treatment having the lowest concentration of phenolics (Table 4). The greatest pressing pressure (0.8–1.6 MPa) resulted in the highest concentration of phenolics (283.6 mg/L) in juices (*p* < 0.01), but no significant differences in the concentration of phenolics were observed in corresponding wines.

### 2.4. Bentonite Requirement

**Figure 1:**
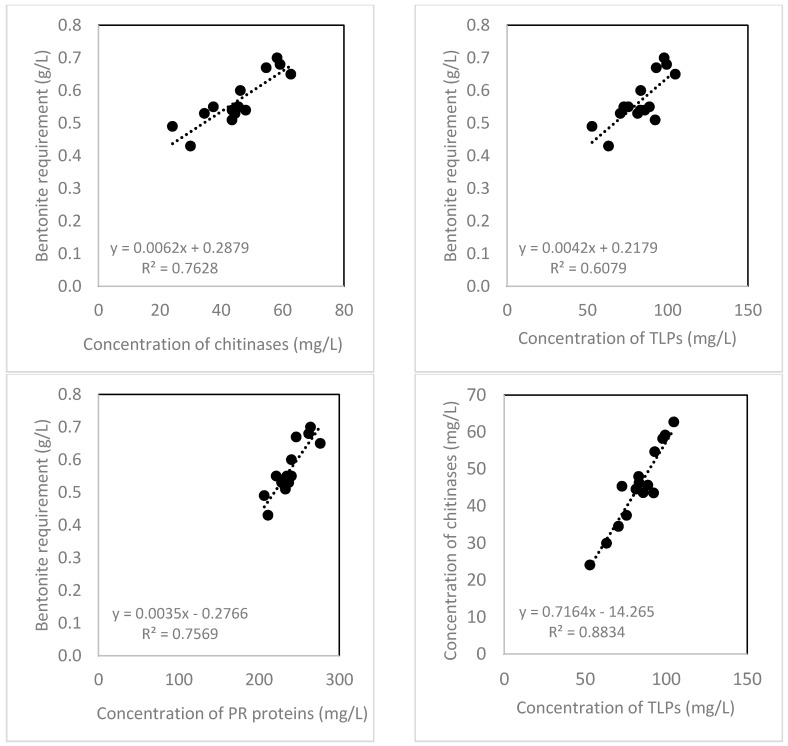
Correlations between bentonite requirement and the concentration of chitinases, TLPs and PR proteins in wines from 2012DP.

A bentonite fining trial was carried out for all wine samples made from combined juice fractions and separate pressing fractions in three different treatments of 2012DP. The bentonite requirement was in a range from 0.43 g/L to 0.70 g/L, and the correlations between bentonite requirement and the concentration of chitinases (R^2^ = 0.76), TLPs (R^2^ = 0.61), and total PR proteins (R^2^ = 0.76) in wine, and the correlation between the concentration of chitinases and TLPs (R^2^ = 0.88) in wine are shown in Figure 1.

## 3. Discussion

Comparing the two hand harvesting treatments (H-WB and H-DC-3), destemming and crushing followed by 3 h skin contact resulted in higher juice yield and greater extraction of proteins and phenolics from grapes into juice, as expected (Table 3). This was reflected in significantly higher concentration of phenolics and chitinases in juices of H-DC-3. No significant difference in the concentration of proteins and TLPs between two treatments was observed, which is likely due to the dilution effect caused by greater juice yield in H-DC-3.

Comparing the H-DC-3 and M-DC-3 treatments, juices from the former had higher concentrations of proteins and TLPs but not phenolics. During machine harvesting, a proportion of grapes leaves, stems and tendrils of grapevines were also collected along with grapes by the machine harvester, which might be expected to result in greater extraction of phenolics from these materials into the resultant juices. However, the concentration of phenolics in juice from M-DC-3 was not significantly different to that from H-DC-3. A decrease in phenolics due to oxidation from juices collected following machine harvester has been observed previously [30]. In addition, the polyphenols extracted from the materials other than grapes (MOG) could interact with proteins and precipitate through the formation of phenolic-protein complexes. Such an interaction between polyphenols and proteins could also explain the lower concentration of proteins observed in juices from M-DC-3. Alternatively, the total amount of extracted proteins, TLPs and chitinases in juices between the H-DC-3 and M-DC-3 treatments were similar, but the juice yield of the M-DC-3 treatment was greater than the H-DC-3 treatment. Thus, the lower concentration of protein in juices from the M-DC-3 treatment might be also partly due to a greater dilution effect.

In an early study [29], which investigated the impact of machine harvesting coupled with long distance transport (20 h) on protein content in juice, it was found the free-run juice from machine harvested grapes had a higher protein content compared to free-run juice obtained by hand-squeezing intact grapes. This was ascribed to increased extraction from grape skins during long distance transport. However, in the study reported here, the concentration of proteins and chitinases in juices between H-WB and M-DC-3 was similar, and the concentration of TLPs was lower in juices of M-DC-3. Although the total amount of extracted proteins was significantly higher in juices from M-DC-3, it was not reflected in the concentration of proteins in juice after taking into account of juice yield. In other work, we have shown that the concentration of proteins in juice with 24 h skin contact was significantly higher than that in juice with 3 h skin contact [31]. Thus, in this study, the extraction of skin proteins with 3 h skin contact was very limited compared to the 20 h transport. In addition, the precipitation of proteins due to the formation of protein-phenolic complex could further counteract the effect of grape skin extraction.

No significant differences were observed for the concentration of phenolics in combined juice fractions obtained under different pressing pressures, but in separate pressing fractions the high pressure resulted in significantly higher concentration of phenolics (Table 4). This was mainly because the initial release of phenolics is from the pulp (predominately hydroxycinnamic acids) and greater pressures and or maceration are required to release phenolics (predominately flavonoids) from skin. However, the juice yield of the high pressure fraction (0.8–1.6 MPa) was very low, and there was no significant increase of the concentration of phenolics in combined juice. Significantly lower concentration of TLPs was observed in juices and wines from high pressure, indicating that TLPs are mainly present in the pulp [9].

The decrease of TLPs and chitinases after fermentation observed in this study is consistent with a previously published study [29,32,33,34] that found around 60% of initial juice PR proteins remained in Sauvignon Blanc wine. In this study, the reduction of chitinases was greater than TLPs, which is likely due to the irreversible denaturation of chitinases. Marangon et al. [35] have shown that chitinases require a lower temperature to unfold, aggregate and precipitate than TLPs, and the denaturation process is irreversible; in contrast, TLPs begin to unfold at a higher temperature, and the denaturation is reversible. In addition, TLPs have different isoforms which have different properties with respect to unfolding temperature with some being heat unstable and forming haze but others not [23,24]. This may explain why bentonite requirement observed in this study had a better positive linear correlation with concentration of chitinases in wine (R^2^ = 0.76) than concentration of TLPs in wine (R^2^ = 0.61), albeit a good positive linear correlation between concentrations of chitinases and TLPs in wine (R^2^ = 0.88). All regressions were statistically significant (*n* = 15, *p* < 0.001). The positive correlation between bentonite requirement and concentration of chitinases observed in this study was also in agreement with a previous study [16] in which the chitinases concentration has been found directly correlated to the turbidity of heat-induced haze formation at 50 °C for 3 h.

## 4. Materials and Methods

### 4.1. Source of Grapes and Corresponding Juice and Wine Samples

Sauvignon Blanc grapes (clone/rootstock: MS/SO4) were sourced from two vineyards located in Marlborough, New Zealand which are recognized to produce wines with different flavor profiles. Dillons Point vineyard located in the Lower Wairau Valley was used in 2011 and 2012, and Ben Morven vineyard located in the Southern Valleys was included in 2012. Each combination of year and vineyard site were designated as a block: 2011DP, 2012DP and 2012BM. For each block, three different grape processing treatments were evaluated (Table 1). Grapes were first hand-harvested from 13 bays of grapevines within two rows (3 vines per bay with row spacing of 2.4 m). Once hand harvested, a commercial harvester (Selectiv Head 8590, Pellenc Ltd., Pertuis, France) was used to machine harvest the remaining grapes from the same two rows of grapevines. The weight of grapes used in each treatment was shown in Table 1. During destemming and crushing, 80 mg/L of potassium metabisulfite (PMS) was added to avoid oxidation. Juice samples from each processing treatment were collected by using a horizontal pneumatic membrane press (PA8, Siprem International S.p.A., Pesaro, Italy) to sequentially increase the pressure to three different pressure points: 0.4 MPa, 0.8 MPa and 1.6 MPa. Thus, there were three combined pressing fractions (0–0.4 MPa, 0–0.8 MPa and 0–1.6 MPa) collected for each treatment in each block. For 2012DP, two additional separate pressing fractions (0.4–0.8 MPa and 0.8–1.6 MPa) were also collected. Juice yield was calculated by using the volume of juice divided by the weight of grapes. All juice samples (about 3 L for each pressing fraction) were settled at 4 °C for 24 h after addition of Rohavin MX enzyme (AB Enzymes, Darmstadt, Germany) at the rate of 0.03 mL/L, and then divided into 3 aliquots for analysis.

Juice samples collected for 2012DP were fermented in 750 mL of wine bottles according to the standard winemaking protocol for Sauvignon Blanc at Marlborough Research Centre [36]. In brief, juices were inoculated with 250 mg/L of EC1118 yeast and fermented at approximately 12 °C. Once fermentation had completed (residual sugar < 2 g/L), 50 mg/L of potassium metabisulfite was added. The ferments were racked and 3 g/L of potassium bitartrate was added for cold stabilization at −4 °C. Sulfur dioxide concentration was adjusted to maintain molecular SO_2_ of approximately 0.8 mg/L. Wines were further filtered (1.2 µm diameter prefilter, then 0.45 µm diameter membrane bottling filter) before being bottled under screw cap using a nitrogen cover. Bottled wines were stored at 10–12 °C until required for further analysis of protein and phenolic concentration and bentonite requirement.

### 4.2. Analysis of Total Soluble Solids and Acidity

Juice samples were analyzed for total soluble solids (TSS), pH and titratable acidity (TA). The TSS and pH were determined using refractometer (MeasumaX, Christchurch, NewZealand) and pH meter (Metrohm AG, Riverview, MI, USA), respectively. The TA was measured on a 5 mL juice sample using an autotitrator (DL50, Metler-Toledo Ltd., Hamilton, New Zealand). Each 5 mL aliquot of juice was diluted with 30 mL distilled water and titrated using 0.1 M NaOH to pH 8.2, with titratable acidity expressed as g/L tartaric acid equivalent.

### 4.3. Determination of Total Proteins

Protein concentration was determined using the EZQ protein quantification kit (Invitrogen, New Zealand) following the manufacturer’s instructions. The calibration curve was carried out using serial dilution of ovalbumin (0–500 mg/L) included in the kit. Fluorescence measurements were taken using excitation/emission settings of 485/590 nm with a 96-well micro-plate reader (FLUOstar Omega, BMG LABTECH, Ortenberg, Germany).

### 4.4. Determination of Total Phenolics

The concentration of total phenolics was determined using a micro scale protocol for the Folin–Ciocalteau colorimetric reaction method [37]. Total phenolics were quantified against a gallic acid standard curve (0 to 500 mg/L). The absorbance readings were taken at 765 nm on a Unicam Heλios UV-VIS Spectrophotometer (Pye Unicam, Cambridge, UK).

### 4.5. Pathogenesis-Related Proteins Analysis by RP-HPLC

The method described in Marangon et al. [33] was used to determine the concentration of PR proteins in juices and wines by reverse-phase (RP) HPLC. Juice and wine samples (50 µL) were loaded at 1 mL/min onto a C8 column (4.6 × 250 mm, Vydac 208TP54, Grace Davison Discovery Sciences, Baulkham, Australia), fitted with a C8 guard column kit (4.6 × 5 mm, Vydac 208GK54, Grace Davison Discovery Sciences, Baulkham, Australia) which was equilibrated in a mixture of 83% (*v*/*v*) solvent B (0.1% trifluoroacetic acid (TFA) in 8% acetonitrile) and 17% solvent A (80% acetonitrile, 0.1% (*v*/*v*) TFA) and held at 35 °C. In this study, the peaks eluting between 9 and 12 min were assigned as TLPs and the peaks eluting between 18 and 25 min were assigned as chitinases [33,38,39]. Quantification of TLPs and chitinases was conducted by comparison with the peak area of thaumatin from *Thaumatococcus daniellii* (Sigma-Aldrich, Auckland, New Zealand), and thus the protein concentration was expressed as thaumatin equivalent.

### 4.6. Bentonite Requirement

Bentonite requirement of the wines was determined using the heat test [40]. A 5% *w*/*v* of stock slurry was prepared by mixing 5 g of bentonite (BDH Laboratory Supplies, Dubai, UAE) in hot water to make 100 mL total. This mixture was stirred overnight until smooth and free of clots. An appropriate volume of stock slurry was added into portions of wine to make linear incremental additions from 0 to 1 g/L with an increment of 0.25 g/L. Wine samples added with bentonite were mixed and centrifuged followed by heating at 80 °C for 6 h, and then held at 4 °C overnight. Haze measurement was carried out on a micro-scale trial (5 mL of wine) using a Unicam Heλios UV-VIS spectrophotometer (Pye Unicam, Cambridge, UK) with absorbance at 520 nm in a 1 mL cuvette [41]. Samples are considered unstable when the difference in absorbance between the bentonite treated sample and a filtered (0.45 µm membrane filter, Sartorius Stedim Biotech, Goettingen, Germany), unheated control is greater than 0.02 absorbance units (au). The bentonite requirement was determined by linear interpolation between the two closest relevant bentonite rates.

### 4.7. Statistical Analysis

All statistical analyses were performed using GenStat v16 software (VSN International Ltd, Hemel Hempstead, UK). For response variables measured across all three blocks, the effects of processing method and extraction pressure (and the interaction terms) were assessed using ANOVA, with block treated as a random factor. Further separation of levels of factors which proved statistically significant (*p* < 0.05) was performed using a post hoc Tukey test.

For examination of the effects of processing method and separate pressing fractions on juice and wine components for the 21012DP block harvest, ANOVA was again used but this time the interaction term could not be included in the analysis due to the lack of replicates for each treatment combination (see Table 4).

The relationship between bentonite requirement and the concentration of chitinases in juice and wine was assessed using simple linear regression.

## 5. Conclusions

Combinations of harvesting and grape processing conditions have great impact on extraction of grape proteins and phenolics into juice, which could consequently affect wine heat stability. The concentration of phenolics in juice is generally increased by machine harvesting, destemming and crushing, 3 h skin contact, and high pressing pressure. However, the concentration of proteins in juice is more complicated, as it is greatly influenced by juice yield and interactions with polyphenols. These results also suggested that consideration should be given to measurement of the concentration of chitinases as a tool to predict bentonite requirement in preference to the time-consuming heat stability test, but a wider range of wines should be tested in the future to validate the feasibility of using the concentration of chitinases as an indicator of bentonite requirement.

## Figures and Tables

**Table 1:** Description of three different grape processing treatments.

Process	Grape Weight	Harvesting Method	Grape Processing	Skin Contact
H-WB	300 kg	Hand	Whole bunch press	0 h
H-DC-3	300 kg	Hand	Destemmed and crushed	3 h
M-DC-3	400 kg	Machine	Destemmed and crushed	3 h

**Table 2:** Analysis of total soluble solids and acidity in grapes harvested in three blocks (*n* = 3).

Block	Process	°Brix	pH	TA (g/L) *
2011DP	H-WB	19.2 ± 0.23	2.95 ± 0.02	12.2 ± 0.02
	H-DC-3	19.6 ± 0.25	3.06 ± 0.02	11.6 ± 0.41
	M-DC-3	20.7 ± 0.29	3.08 ± 0.01	11.1 ± 0.30
2012DP	H-WB	19.6 ± 0.32	2.98 ± 0.01	14.6 ± 0.24
	H-DC-3	19.6 ± 0.25	3.06 ± 0.02	13.1 ± 0.13
	M-DC-3	19.9 ± 0.23	3.08 ± 0.01	13.0 ± 0.05
2012BM	H-WB	21.6 ± 0.23	2.94 ± 0.01	12.3 ± 0.36
	H-DC-3	21.4 ± 0.23	3.03 ± 0.02	11.9 ± 0.17
	M-DC-3	21.7 ± 0.32	3.07 ± 0.04	11.4 ± 0.58

* Titratable acidity, expressed as tartaric acid equivalent.

**Table 3:** Statistical analysis of juices from three blocks.

Source of Variation	Juice Yield (%)	Concentration of Each Component in Juice (mg/L)				Extraction of Each Component in Juice (mg/kg of Grapes) ^1^			
		Proteins *	Phenolics ^#^	TLPs ^†^	Chitinases ^†^	Proteins *	Phenolics ^#^	TLPs ^†^	Chitinases ^†^
Process									
H-WB	39.3 a	131.5 ab	204.2 a	106.6 b	75.9 a	52.1 a	78.4 a	41.8 a	30.3 a
H-DC-3	59.0 b	151.3 b	246.3 b	107.3 b	83.2 b	88.6 b	144.6 b	63.1 b	48.9 b
M-DC-3	64.5 c	120.0 a	235.5 b	99.0 a	76.7 ab	76.5 b	151.9 b	63.7 b	49.3 b
Pressure									
0–0.4 MPa	39.4 a	139.3 a	239.6 a	104.3 ab	76.5 a	56.4 a	97.1 a	41.2 a	31.0 a
0–0.8 MPa	57.0 b	133.5 a	227.6 a	107.6 b	81.2 a	74.9 b	131.7 b	60.5 b	45.9 b
0–1.6 MPa	66.3 c	130.0 a	218.9 a	101.0 a	78.1 a	85.8 b	146.1 b	66.8 b	51.6 b
Process * Pressure									
H-WB 0–0.4 MPa	15.3 a	123.5 a	217.3 a	102.4 a	68.7 a	18.5 a	32.9 a	15.4 a	10.3 a
H-WB 0–0.8 MPa	42.6 b	139.6 a	199.0 a	114.5 a	81.5 a	59.3 ab	84.7 b	48.6 b	34.6 b
H-WB 0–1.6 MPa	59.9 d	131.3 a	196.4 a	102.9 a	77.4 a	78.4 b	117.6 bc	61.5 bc	46.1 bc
H-DC 3 0–0.4 MPa	49.0 c	162.2 a	257.9 a	109.5 a	83.7 a	80.0 b	126.5 bcd	53.8 bc	41.2 bc
H-DC 3 0–0.8 MPa	60.8 d	148.8 a	252.1 a	109.6 a	85.2 a	90.3 b	153.3 cd	66.6 bc	51.7 bc
H-DC 3–1.6 MPa	67.1 e	142.9 a	228.8 a	102.7 a	80.7 a	95.4 b	154.1 cd	68.9 bc	53.8 bc
M-DC 3–0.4 MPa	54.0 c	132.1 a	243.4 a	100.9 a	77.1 a	70.9 b	131.8 cd	54.4 bc	41.5 bc
M-DC 3 0–0.8 MPa	67.6 e	112.2 a	231.7 a	98.8 a	76.8 a	75.3 b	157.1 cd	66.4 bc	51.5 bc
M-DC 3 0–1.6 MPa	71.9 e	115.8 a	231.5 a	97.2 a	76.1 a	83.5 b	166.6 d	70.1 c	54.9 c

^1^ Extracted amount of each component is calculated by multiplying juice yield and corresponding concentration. * protein concentration was determined by EZQ kit, which is expressed as ovalbumin equivalent; ^#^ phenolics was determined by Folin–Ciocalteau method, which is expressed as gallic acid equivalent; ^†^ TLPs and chitinases were determined by RP-HPLC, which is expressed as thaumatin equivalent. Statistically significant differences (*p* < 0.05) between treatments using a post hoc Tukey test are indicated by different letters.

**Table 4:** Statistical analysis of juices and wines obtained from the separate pressing fractions in 2012DP.

Source of Variation	Concentration of Each Component in Juice (mg/L)				Concentration of Each Component in Wine (mg/L)			
	Proteins *	Phenolics ^#^	TLPs ^†^	Chitinases ^†^	Proteins *	Phenolics ^#^	TLPs ^†^	Chitinases ^†^
Process								
H-WB	173.0 a	234.5 a	140.9 a	102.7 a	99.4 a	206.5 a	89.9 a	50.5 a
H-DC-3	180.0 a	278.3 b	133.9 a	103.6 a	94.9 a	232.1 a	80.8 a	44.2 a
M-DC-3	101.0 a	277.0 b	123.1 a	102.0 a	79.4 a	238.1 a	70.7 a	34.7 a
Pressure								
0–0.4 MPa	170.0 a	257.1 a	137.6 ab	99.2 a	97.9 a	220.9 a	87.5 ab	44.6 a
0.4–0.8 MPa	173.0 a	249.0 a	144.3 b	112.0 a	103.3 a	214.6 a	91.1 b	51.8 a
0.8–1.6 MPa	111.0 a	283.6 b	116.0 a	97.0 a	72.5 a	241.1 a	63.0 a	33.1 a

* Protein concentration was determined by EZQ kit, which is expressed as ovalbumin equivalent; ^#^ phenolics was determined by Folin–Ciocalteau method, which is expressed as gallic acid equivalent; ^†^ TLPs and chitinases were determined by RP-HPLC, which is expressed as thaumatin equivalent. Statistically significant differences (*p* < 0.05) between treatments using a post hoc Tukey test are indicated by different letters.

## Supplementary Materials

Supplementary Materials are available online, Table S1: Compositional analysis of juices and wines from different treatments across the three blocks.
