# Supplementary material for: Extraction of Pathogenesis-Related Proteins and Phenolics in Sauvignon Blanc as Affected by Grape Harvesting and Processing Conditions
Source: Molecules. 2017 Jul 12;22(7):1164. doi: 10.3390/molecules22071164 (PMC6152371; doi:10.3390/molecules22071164)
Supplement: Supplementary file 1 [file molecules-22-01164-s001.pdf]

**Supplementary Material**

# Extraction of pathogenesis-related proteins and phenolics in Sauvignon Blanc as affected by grape harvesting and processing conditions

**Bin Tian [1,*], Roland Harrison [1], James Morton [1], Marlene Jaspers [1], Simon Hodge [1], Claire Grose [2] and Mike Trought [2]**

**Table S1.** Compositional analysis of juices and wines from different treatments across the three blocks ($n$ = 3).

| Block | Process | Pressure (Mpa) | Juice Yield (%) | Concentration of Each Component in Juice (mg/L) | | | | Extraction of Each Component in Juice (mg/kg of Grapes) [1] | | | | Concentration of Each Component in Wine (mg/L) | | | |
|---|---|---|---|---|---|---|---|---|---|---|---|---|---|---|---|
| | | | | Proteins * | Phenolics ♯ | TLPs † | Chitinases † | Proteins | Phenolics | TLPs | Chitinases | Proteins * | Phenolics ♯ | TLPs † | Chitinases † |
| 2011DP | H-WB | 0–0.4 | 13.5 | 148.3 ± 38.4 | 248.8 ± 34.7 | 113.3 ± 3.4 | 83.2 ± 1.6 | 20.0 ± 5.2 | 33.6 ± 4.7 | 15.3 ± 0.5 | 11.2 ± 0.2 | n/a | n/a | n/a | n/a |
| | | 0–0.8 | 42.2 | 159.1 ± 32.7 | 195.0 ± 36.1 | 118.3 ± 2.2 | 92.5 ± 0.6 | 67.1 ± 13.8 | 82.3 ± 15.2 | 49.9 ± 0.9 | 39.0 ± 0.2 | n/a | n/a | n/a | n/a |
| | | 0–1.6 | 55.7 | 141.0 ± 33.1 | 195.5 ± 25.9 | 108.5 ± 1.4 | 89.7 ± 0.8 | 78.6 ± 18.4 | 108.9 ± 14.4 | 60.4 ± 0.8 | 50.0 ± 0.4 | n/a | n/a | n/a | n/a |
| | H-DC-3 | 0–0.4 | 50.0 | 224.4 ± 37.4 | 268.3 ± 35.1 | 119.0 ± 1.3 | 107.0 ± 0.4 | 112.2 ± 18.7 | 134.2 ± 17.5 | 59.5 ± 0.7 | 53.5 ± 0.2 | n/a | n/a | n/a | n/a |
| | | 0–0.8 | 60.0 | 192.5 ± 13.9 | 242.2 ± 13.6 | 114.6 ± 2.3 | 105.1 ± 2.4 | 115.5 ± 8.4 | 145.3 ± 8.1 | 68.8 ± 1.4 | 63.1 ± 1.5 | n/a | n/a | n/a | n/a |
| | | 0–1.6 | 62.7 | 175.3 ± 30.4 | 211.2 ± 27.1 | 112.0 ± 0.8 | 102.2 ± 1.6 | 109.9 ± 19.1 | 132.4 ± 17.0 | 70.2 ± 0.5 | 64.1 ± 1.0 | n/a | n/a | n/a | n/a |
| | M-DC-3 | 0–0.4 | 50.4 | 153.8 ± 38.6 | 217.8 ± 39.3 | 98.1 ± 5.3 | 83.1 ± 4.9 | 77.5 ± 19.5 | 109.8 ± 19.8 | 49.4 ± 2.7 | 41.9 ± 2.5 | n/a | n/a | n/a | n/a |
| | | 0–0.8 | 63.9 | 121.9 ± 7.2 | 196.3 ± 11.2 | 102.2 ± 2.2 | 87.6 ± 1.7 | 77.9 ± 4.6 | 125.4 ± 7.1 | 65.3 ± 1.4 | 56.0 ± 1.1 | n/a | n/a | n/a | n/a |
| | | 0–1.6 | 71.5 | 118.8 ± 15.7 | 180.1 ± 15.2 | 98.5 ± 0.8 | 85.4 ± 0.8 | 85.0 ± 11.3 | 128.8 ± 10.9 | 70.4 ± 0.6 | 61.1 ± 0.6 | n/a | n/a | n/a | n/a |
| 2012DP | H-WB | 0–0.4 | 15.7 | 170.7 ± 18.7 | 228.0 ± 1.7 | 138.8 ± 4.6 | 94.1 ± 4.1 | 26.8 ± 2.9 | 35.8 ± 0.3 | 21.8 ± 0.7 | 14.8 ± 0.6 | 87.5 ± 5.9 | 203.0 ± 5.9 | 92.2 ± 3.8 | 43.5 ± 1.4 |
| | | 0.4-0.8 | 26.6 | 205.5 ± 6.4 | 224.6 ± 3.8 | 160.2 ± 3.1 | 116.2 ± 0.4 | 54.7 ± 1.7 | 59.8 ± 1.0 | 42.6 ± 0.8 | 30.9 ± 0.1 | 120.2 ± 20.1 | 202.7 ± 1.7 | 104.8 ± 3.2 | 62.7 ± 3.4 |
| | | 0–0.8 | 42.3 | 181.0 ± 11.2 | 222.4 ± 0.8 | 152.0 ± 2.0 | 110.0 ± 0.5 | 76.6 ± 4.7 | 94.1 ± 0.4 | 64.3 ± 0.9 | 46.5 ± 0.2 | 107.3 ± 7.4 | 201.9 ± 4.3 | 99.4 ± 5.2 | 59.2 ± 3.4 |
| | | 0.8-1.6 | 19.6 | 144.3 ± 6.6 | 250.8 ± 8.7 | 123.6 ± 1.1 | 97.9 ± 0.8 | 28.3 ± 1.3 | 49.1 ± 1.7 | 24.2 ± 0.2 | 19.2 ± 0.2 | 90.4 ± 11.1 | 213.8 ± 3.4 | 72.8 ± 3.7 | 45.3 ± 3.4 |
| | | 0–1.6 | 61.9 | 181.1 ± 5.6 | 232.1 ± 3.5 | 140.8 ± 3.2 | 105.6 ± 1.5 | 112.1 ± 3.5 | 143.7 ± 2.1 | 87.1 ± 2.0 | 65.4 ± 0.9 | 116.6 ± 12.3 | 209.1 ± 0.8 | 92.9 ± 3.9 | 54.7 ± 2.1 |
| | H-DC-3 | 0–0.4 | 49.0 | 182.5 ± 13.8 | 275.8 ± 1.7 | 136.9 ± 4.0 | 100.2 ± 2.4 | 89.4 ± 6.8 | 135.1 ± 0.8 | 67.1 ± 1.9 | 49.1 ± 1.2 | 103.7 ± 2.8 | 236.9 ± 3.2 | 81.4 ± 4.4 | 44.5 ± 3.2 |
| | | 0.4-0.8 | 12.3 | 201.2 ± 20.4 | 259.1 ± 0.8 | 148.6 ± 3.3 | 115.6 ± 1.7 | 24.7 ± 2.5 | 31.9 ± 0.1 | 18.3 ± 0.4 | 14.2 ± 0.2 | 114.1 ± 10.2 | 219.4 ± 1.3 | 97.9 ± 8.2 | 58.2 ± 3.9 |
| | | 0–0.8 | 61.3 | 162.4 ± 19.9 | 272.7 ± 1.0 | 138.0 ± 3.5 | 102.4 ± 2.7 | 99.6 ± 12.2 | 167.2 ± 0.6 | 84.6 ± 2.1 | 62.8 ± 1.6 | 90.9 ± 11.5 | 230.2 ± 3.9 | 83.3 ± 5.6 | 46.3 ± 4.9 |
| | | 0.8-1.6 | 8.6 | 157.3 ± 12.1 | 300.2 ± 2.4 | 116.3 ± 4.3 | 94.9 ± 0.7 | 13.5 ± 1.0 | 25.8 ± 0.2 | 10.0 ± 0.4 | 8.2 ± 0.1 | 66.9 ± 8.7 | 240.2 ± 2.9 | 63.2 ± 10.8 | 30.0 ± 7.4 |
| | | 0–1.6 | 69.9 | 182.4 ± 11.7 | 274.6 ± 0.5 | 134.7 ± 4.1 | 100.8 ± 2.5 | 127.5 ± 8.2 | 192.0 ± 0.3 | 94.2 ± 2.9 | 70.5 ± 1.7 | 95.9 ± 11.2 | 211.9 ± 7.9 | 83.0 ± 5.6 | 48.0 ± 3.7 |
| | M-DC-3 | 0–0.4 | 55.3 | 158.1 ± 9.0 | 267.7 ± 1.3 | 137.0 ± 4.3 | 103.4 ± 1.6 | 87.5 ± 5.0 | 148.0 ± 0.7 | 75.8 ± 2.4 | 57.2 ± 0.9 | 102.4 ± 6.0 | 223.0 ± 11.4 | 88.7 ± 2.0 | 45.6 ± 5.3 |
| | | 0.4-0.8 | 12.9 | 112.7 ± 42.0 | 263.3 ± 7.3 | 124.0 ± 2.9 | 104.3 ± 0.7 | 14.5 ± 5.4 | 34.0 ± 0.9 | 16.0 ± 0.4 | 13.4 ± 0.1 | 75.6 ± 10.4 | 221.9 ± 0.5 | 70.5 ± 2.4 | 34.5 ± 1.8 |
| | | 0–0.8 | 68.2 | 158.3 ± 12.7 | 269.1 ± 2.2 | 133.3 ± 5.1 | 102.9 ± 0.8 | 108.0 ± 8.7 | 183.5 ± 1.5 | 90.9 ± 3.5 | 70.2 ± 0.6 | 95.8 ± 10.6 | 229.6 ± 2.9 | 85.8 ± 5.0 | 43.6 ± 3.6 |
| | | 0.8-1.6 | 4.8 | 32.8 ± 4.1 | 299.9 ± 7.1 | 108.2 ± 2.1 | 98.3 ± 0.7 | 1.6 ± 0.2 | 14.4 ± 0.3 | 5.2 ± 0.1 | 4.7 ± 0.0 | 60.2 ± 15.2 | 269.4 ± 4.6 | 53.0 ± 8.5 | 24.1 ± 5.5 |
| | | 0–1.6 | 73.0 | 164.8 ± 10.4 | 272.1 ± 1.7 | 132.4 ± 4.3 | 102.3 ± 0.6 | 120.3 ± 7.6 | 198.7 ± 1.3 | 96.6 ± 3.1 | 74.7 ± 0.5 | 84.7 ± 9.7 | 220.8 ± 6.6 | 75.6 ± 5.4 | 37.5 ± 1.1 |
| 2012BM | H-WB | 0–0.4 | 16.7 | 51.5 ± 8.8 | 175.2 ± 9.0 | 55.3 ± 1.4 | 28.7 ± 2.4 | 8.6 ± 1.5 | 29.3 ± 1.5 | 9.2 ± 0.2 | 4.8 ± 0.4 | n/a | n/a | n/a | n/a |
| | | 0–0.8 | 43.3 | 78.7 ± 3.6 | 179.5 ± 8.1 | 73.1 ± 1.2 | 42.0 ± 0.1 | 34.1 ± 1.6 | 77.7 ± 3.5 | 31.6 ± 0.5 | 18.2 ± 0.1 | n/a | n/a | n/a | n/a |
| | | 0–1.6 | 62.0 | 71.8 ± 2.7 | 161.6 ± 25.1 | 59.4 ± 7.1 | 36.8 ± 3.7 | 44.5 ± 1.7 | 100.2 ± 15.5 | 36.8 ± 4.4 | 22.8 ± 2.3 | n/a | n/a | n/a | n/a |
| | H-DC-3 | 0–0.4 | 48.0 | 79.8 ± 11.9 | 229.8 ± 20.1 | 72.6 ± 6.8 | 44.0 ± 3.4 | 38.3 ± 5.7 | 110.3 ± 9.7 | 34.8 ± 3.2 | 21.1 ± 1.6 | n/a | n/a | n/a | n/a |
| | | 0–0.8 | 61.0 | 91.5 ± 42.0 | 241.6 ± 8.2 | 76.1 ± 5.2 | 48.0 ± 3.0 | 55.8 ± 25.6 | 147.4 ± 5.0 | 46.4 ± 3.2 | 29.3 ± 1.9 | n/a | n/a | n/a | n/a |
| | | 0–1.6 | 68.7 | 71.0 ± 16.0 | 200.7 ± 11.4 | 61.5 ± 5.9 | 39.1 ± 4.2 | 48.8 ± 11.0 | 137.9 ± 7.8 | 42.2 ± 4.0 | 26.9 ± 2.9 | n/a | n/a | n/a | n/a |
| | M-DC-3 | 0–0.4 | 56.3 | 84.5 ± 10.9 | 244.6 ± 13.2 | 67.5 ± 3.9 | 44.9 ± 2.1 | 47.6 ± 6.1 | 137.7 ± 7.4 | 38.0 ± 2.2 | 25.3 ± 1.2 | n/a | n/a | n/a | n/a |
| | | 0–0.8 | 70.7 | 56.4 ± 11.6 | 229.8 ± 8.1 | 60.8 ± 2.2 | 39.8 ± 0.7 | 39.9 ± 8.2 | 162.4 ± 5.8 | 43.0 ± 1.5 | 28.2 ± 0.5 | n/a | n/a | n/a | n/a |
| | | 0–1.6 | 71.2 | 63.7 ± 7.9 | 242.2 ± 7.4 | 60.9 ± 1.1 | 40.6 ± 0.8 | 45.3 ± 5.6 | 172.4 ± 5.3 | 43.4 ± 0.8 | 28.9 ± 0.6 | n/a | n/a | n/a | n/a |

[1] extracted amount of each component is calculated by multiplying juice yield and corresponding concentration. * protein concentration was determined by EZQ kit, which is expressed as ovalbumin equivalent; ♯ phenolics was determined by Folin-Ciocalteau method, which is expressed as gallic acid equivalent; † TLPs and chitinases were determined by RP-HPLC, which is expressed as thaumatin equivalent. n/a, juices were not bottle fermented to get wines.
